# The ethical climate in paediatric oncology—A national cross‐sectional survey of health‐care personnel

**DOI:** 10.1002/pon.5009

**Published:** 2019-02-14

**Authors:** Pernilla Pergert, Cecilia Bartholdson, Margareta af Sandeberg

**Affiliations:** ^1^ Childhood Cancer Research Unit, Department of Women's & Children's Health Karolinska Institutet Stockholm Sweden; ^2^ Paediatric Haematology and Oncology, Children's and Women's Health Care Karolinska University Hospital Stockholm Sweden; ^3^ Paediatric Neurology and Muscular Skeletal Disorders and Homecare, Children's and Women's Health Care Karolinska University Hospital Stockholm Sweden

**Keywords:** cancer; ethical climate; ethics, institutional; health‐care survey; medical staff, hospital; nursing staff, hospital; oncology; paediatric oncology; personnel, hospital

## Abstract

**Objective:**

To describe health‐care personnel's (HCP's) perceptions of the ethical climate at their workplace in paediatric oncology.

**Methods:**

A cross‐sectional survey was conducted using the Swedish version of the shortened Hospital Ethical Climate Survey (HECS‐S). HCP at all six paediatric oncology centres (POCs) in Sweden were invited to participate. Analysis included descriptive statistics, the Mann‐Whitney U test (differences between groups) and Spearman's rank correlation. Informed consent was assumed when the respondents returned the survey.

**Results:**

A high response rate was achieved as 278 HCP answered the questionnaire. Medical doctors perceived the ethical climate to be more positive than registered nurses and nursing assistants. At the POC with the significantly lowest values concerning immediate manager, no significant correlation with the other items was found. At the POC with the poorest ethical climate, HCP also had the lowest perception of the possibility of practicing ethically good care.

**Conclusions:**

Differences between centres and professional groups have been demonstrated. A negative perception of the immediate manager does not necessarily mean that the ethical climate is poor, but the manager's ability to provide the conditions for an open dialogue within the health‐care team is key to achieving an ethical climate.

## 1 BACKGROUND

Paediatric oncology includes highly advanced medical and nursing care and is still the most common cause of mortality in children up to 15 years of age.^1^ It involves difficult ethical issues including deciding on the treatment level, for example, balancing pain relief and ending curative treatment,^2^ perceived to be extremely difficult.^3^ “Ethical climate” describes the workplace environment and refers to health‐care personnel's (HCP's) shared perceptions of the organisation that influences behaviours and attitudes, including how ethical issues are handled.^4^ A management that supports ethical behaviour is likely to have favourable organisational outcomes.^5^ An ethical climate is characterised by teamwork, a shared vision of care, and support.^6^ Research of the ethical climate in nursing has been performed for decades and has led to extensive knowledge.^7^ Studies have shown that the ethical climate influences moral distress and job satisfaction of nurses, as well as the quality of patient care.^8^, ^9^, ^10^, ^11^ More recently, some research has also included medical doctors' (MDs') perceptions of the ethical climate.^12^ A study of the ethical climate amongst HCP caring for children with cancer in a Swedish hospital showed that only one third felt that they were able to practice ethically good care, and nurses were generally less positive than MDs.^13^ Dzeng et al propose that the ethical climate, as a part of the sociocultural context, is crucial to the quality of end‐of‐life care.^14^ A previous study has revealed the importance of systemic factors—in particular, the hospital ethical climate—on inappropriately aggressive treatment at end of life.^15^ An ethical climate enhances the ability of meeting the care needs of patients and families.^8^ Thus, the focus on organisational structures and the ethical climate rather than only on the individual characteristics of the HCP is essential in order to gain knowledge of how to best support and enable HCP in providing ethically good care. Therefore, the objectives of this study were to describe HCP's perceptions of the ethical climate at their workplace in paediatric oncology and to identify differences between groups (professions, genders, years of experience, and centres).

## 2 METHODS

A quantitative cross‐sectional survey was conducted using a Swedish translation^16^ of the shortened version^17^ of Olson's^4^ Hospital Ethical Climate Survey (HECS‐S). The settings were all six Swedish paediatric oncology centres (POCs) located at university hospitals in Lund, Gothenburg, Linköping, Stockholm, Uppsala, and Umeå. All major professional groups involved in direct patient care, including registered nurses (RNs, n = 167), MDs (n = 70), and nursing assistants (NAs, n = 72), were invited to participate in this national multisite study (n = 309).

### 2.1 Instrument

The paper survey included demographic questions, as well as the above‐mentioned Swedish HECS‐S, an instrument for assessing HCP's perceptions of the ethical climate. The Swedish HECS‐S includes all 14 items from the original HECS‐S as well as items added to accomplish a multi‐professional instrument relevant for paediatrics (Table 1).^16^ Respondents were asked to state how often they perceived the statements to be consistent with the situation at their workplace on a five‐point Liker‐type scale with the labels “Almost never—Almost always.”

**Table 1:** Dimensions and shortened items of the Swedish HECS‐S

Dimensions	Shortened items
***HCP's relationship with:***	
• The hospital	Hospital's values shared
	Hospital guidelines help
• The immediate manager	My immediate manager helps me decide in patient care
	My immediate manager I trust
	My immediate manager helps my co‐workers decide in patient care
• The patients/parents	Patients' wishes taken into account
	Parents' wishes taken into account
***Relationship between:***	
• MD/RN	MDs and RNs trust one another
	MDs and RNs respect each other's opinions
	MDs ask RNs about their opinion
• RN/NA	RNs and NAs trust one another
	RNs and NAs respect each other's opinions
	RNs ask NAs about their opinions
***Team interactions***	My co‐workers listen
	Feelings and values of everyone considered
	Conflicts openly dealt with
	Competent co‐workers
	Openness asking questions, learning
***Identifying and dealing with ethical issues***	Ethical issues identified
	We talk about dealing with ethical issues
***Care as it should be practiced***	Practice care as I think it should be

Abbreviations: HCP, health‐care personnel; MD, medical doctor; NA, nurs‐ing assistant; RN, registered nurse.

### 2.2 Data collection

Data collection was conducted during meetings/training sessions arranged by each of the centres. The local coordinator subsequently invited HCP who were unable to attend these sessions to answer the survey and sent those by mail. Data collection was conducted from February to September 2016.

### 2.3 Data analysis

All statistical analyses were conducted using the Statistical Package for Social Sciences version 25.0. Descriptive statistics (frequencies, mean values, and SD) were calculated, and differences in distribution between groups were tested using the Mann‐Whitney U test. Correlations between per person mean values of (groups of) items were tested using Spearman's rank correlation. *P* values less than 0.05 were considered statistically significant. Up to 10% missing items were considered acceptable.^18^

### 2.4 Ethical considerations

The research team provided oral information at the sessions and written information together with the survey. The information included the aim of the study and when the respondents returned the survey informed consent was assumed. The total number of respondents per centre will not be disclosed, and the results are reported in a way that protects the integrity of the centres. In its advisory statement, the Regional Ethical Review Board in Stockholm had no ethical objections (D‐no: 2015/1782‐31/5).

## 3 RESULTS

### 3.1 Respondents

HCP (n = 278) from six POCs (henceforth referred to with random letters) answered the questionnaire, response rate 89%. The mean number of respondents from each of the POCs was 47, range 29 to 74. The demographic characteristics of respondents is presented in Table 2.

**Table 2:** Demographic characteristics of respondents

Professional Groups n = 278		Genders n = 278		Years Working in Paediatrics n = 277	
		Female	Male	<5 y	≥5 y
	n (%)	n (%)	n (%)	n (%)	n (%)
All health‐care personnel		230 (83)	48 (17)	83 (30)	194 (70)
RNs	157 (56)	142 (90)	15 (10)	58 (37)	98 (62)
MDs	55 (20)	26 (47)	29 (53)	2 (4)	53 (96)
NAs	66 (24)	62 (94)	4 (6)	23 (35)	43 (65)

Abbreviations: MD, medical doctor; NA, nursing assistant; RN, registered nurse.

The overall highest and lowest scored items and differences between groups are presented below.

### 3.2 Overall highest and lowest scored items in the HECS‐S

The five items with the highest overall values included three items concerning team interactions and the two items concerning patients/parents. The five items with the lowest overall values were the two hospital items, two items on team interactions and, finally, the item on MDs asking RNs for their opinions regarding treatment (Table 3).

**Table 3:** Mean value and SD on all items for the whole group and for each of the professional groups, and *P* values when significant differences were found between groups

Shortened Items (Number of Respondents)	Overall n = 278	RNs n = 157	MDs n = 55	NAs n = 66	RNs vs MDs	RNs vs NAs	MDs vs NAs
	Mean (SD)	Mean (SD)	Mean (SD)	Mean (SD)	*P* Value		
Coworkers listen (n = 276)	4.53 (0.61)^a^	4.51 (0.62)	4.76 (0.47)	4.38 (0.65)	0.007		<0.001
Manager helps (n = 274)	3.77 (1.28)	3.51 (1.33)	4.54 (0.75)	3.74 (1.28)	<0.001		<0.001
Hospital guidelines help (n = 266)	2.97 (0.96)^b^	2.87 (0.94)	2.83 (0.94)	3.31 (0.98)		0.005	0.011
MDs and RNs trust (n = 275)	4.25 (0.74)	4.24 (0.73)	4.53 (0.54)	4.05 (0.83)	0.013		0.001
RNs and NAs trust (n = 267)	3.98 (0.93)	3.87 (1.04)	4.22 (0.74)	4.09 (0.70)			
Hospital's values shared (n = 272)	3.51 (0.94)^b^	3.42 (0.98)	3.51 (0.91)	3.74 (0.81)		0.028	
Feelings and values considered (n = 274)	3.51 (0.91)^b^	3.43 (0.92)	3.62 (0.89)	3.60 (0.88)			
Manager I trust. (n = 276)	4.25 (0.96)	4.13 (0.99)	4.75 (0.52)	4.05 (1.02)	<0.001		<0.001
Conflicts openly dealt with (n = 272)	3.29 (1.02)^b^	3.18 (1.05)	3.67 (0.88)	3.20 (0.98)	0.002		0.004
MDs and RNs respect opinions (n = 274)	3.92 (0.87)	3.85 (0.92)	4.38 (0.56)	3.68 (0.81)	<0.001		<0.001
RNs and NAs respect opinions (n = 261)	3.78 (0.90)	3.71 (0.96)	4.05 (0.65)	3.80 (0.85)			
Competent coworkers (n = 277)	4.58 (0.58)^a^	4.53 (0.61)	4.89 (0.32)	4.46 (0.59)	<0.001		<0.001
Patients' wishes (n = 276)	4.43 (0.65)^a^	4.40 (0.66)	4.44 (0.66)	4.52 (0.62)			
Parents' wishes (n = 277)	4.47 (0.57)^a^	4.46 (0.59)	4.49 (0.51)	4.49 (0.59)			
Manager helps coworkers (n = 268)	3.75 (1.19)	3.48 (1.23)	4.36 (0.92)	3.89 (1.07)	<0.001	0.024	0.006
Openness asking questions, learning (n = 274)	4.48 (0.71)^a^	4.45 (0.75)	4.60 (0.66)	4.45 (0.67)			
Practice care as it should be (n = 275)	4.16 (0.77)	4.06 (0.82)	4.40 (0.60)	4.20 (0.73)	0.008		
MDs ask RNs (n = 273)	3.36 (1.03)^b^	3.25 (1.10)	3.62 (0.89)	3.40 (0.95)	0.024		
RNs ask NAs (n = 257)	3.65 (0.88)	3.66 (0.88)	3.58 (0.69)	3.66 (0.97)			
Ethical issues identified (n = 276)	3.89 (0.89)	3.72 (0.93)	4.41 (0.71)	3.88 (0.78)	<0.001		<0.001
Dealing ethical issues (n = 277)	3.68 (1.02)	3.49 (1.08)	4.22 (0.85)	3.69 (0.85)	<0.001		0.001

Abbreviations: MD, medical doctor; NA, nursing assistant; RN, registered nurse; SD, standard deviation. Differences tested by the Mann‐Whitney U test.

a One of the five items with the highest overall values.

b One of the five items with the lowest overall values.

### 3.3 Differences between professional groups

MDs scored significantly higher than RNs and NAs on 10 items (Table 3), including the three items regarding the immediate manager and three regarding team interactions, two about the relationship between RNs and MDs, and the two about identifying and dealing with ethical issues. MDs also scored significantly higher than RNs on two items, including asking RNs for their opinions and being able to practice care as they think it should be practiced. Few significant differences were identified between RNs and NAs. NAs scored significantly higher than RNs on the item about shared hospital values and significantly higher than RNs and MDs on the item regarding hospital guidelines.

### 3.4 Gender differences

No gender differences were identified except for two items. Male respondents scored significantly (*P* = 0.041) higher (mean 3.63) than female (mean 3.30) on the item on physicians asking nurses for their opinions. This difference was also present in the group of MDs in which male respondents scored significantly (*P* = 0.002) higher (mean 3.97) than female respondents (mean 3.23). Female respondents scored significantly (*P* = 0.034) higher (mean 3.03) than male (mean 2.67) on the item concerning hospital guidelines. However, there were no significant differences between genders in the professional groups on this item.

### 3.5 Differences between years of experience

In three items, differences were identified between the groups with different levels of experience of paediatrics. Respondents with less than 5 years of experience scored significantly (*P* = 0.010) higher (mean 3.21) on the item concerning being helped by hospital guidelines than those with more experience (mean 2.86). This difference was also significant (*P* ≤0.001) when looking at the group of NAs but not at the group of RNs.

The item concerning giving attention to ethical problems scored significantly (*P* = 0.049) higher for those with 5 years of experience or more (mean 3.97) than those with less experience (mean 3.73). Likewise, the item concerning talking about different ways of dealing with ethical issues scored significantly (*P* = 0.017) higher for those with 5 years of experience or more (mean 3.78) than those with less experience (mean 3.46). However, the difference in these two items was not significant in the groups of NAs and RNs.

### 3.6 Differences between POCs

One of the centres (A) scored significantly lower than the other POCs on the three items regarding the immediate manager and also the item about conflicts being dealt with openly. Unlike the other centres (B: *r* = 0.60, *P* = 0.001; C: *r* = 0.38, *P* = 0.015; D: *r* = 0.64, *P* < 0.001; E: *r* = 0.55, *P* < 0.001; F: *r* = 0.75, *P* < 0.001), no significant correlation of per person mean values between the items regarding immediate manager and the other items was found (A: *r* = 0.28, *P* = 0.124). This centre did not have significantly lower values on any of the other items. However, it had significantly higher scores on four items concerning the relationship between MD/RN (n = 2), team interactions (n = 1), and patient wishes (n = 1) (Table 4).

**Table 4:** Differences in mean values and SD on all items between three different POCs compared with all the other centres and p‐values when significant differences were found between the centres

Shortened Items	POC A Mean (SD)	Other POCs (n = 5) Mean (SD)	*P* Values	POC B Mean (SD)	Other POCs (n = 5) Mean (SD)	*P* Values	POC C Mean (SD)	Other POCs (n = 5) Mean (SD)	*P* Values
Coworkers listen	4.50 (0.68)	4.53 (0.60)		4.54 (0.65)	4.52 (0.60)		4.54 (0.60)	4.53 (0.62)	
Manager helps	2.53 (1.55)	3.92 (1.16)***	<0.001	4.05 (1.08)	3.67 (1.34)*	0.049	3.60 (1.48)	3.80 (1.25)	
Hospital guidelines help	2.83 (0.79	2.98 (0.98)		3.10 (0.98)	2.92 (0.95)		2.79 (1.01)	3.00 (0.95)	
MDs and RNs trust	4.61 (0.72)	4.20 (0.73)**	0.001	4.29 (0.81)	4.23 (0.71)		4.12 (0.68)	4.27 (0.75)	
RNs and NAs trust	3.94 (0.93)	3.98 (0.93)		4.24 (0.73)	3.88 (0.97)**	0.009	3.23 (1.05)	4.11 (0.84)***	<0.001
Hospital's values shared	3.34 (0.94)	3.53 (0.94)		3.64 (1.01)	3.46 (0.91)		3.24 (1.04)	3.55 (0.91)	
Feelings and values considered	3.84 (1.07)	3.47 (0.88)*	0.042	3.51 (0.87)	3.51 (0.92)		3.27 (0.87)	3.55 (0.91)	
Manager I trust	3.35 (1.25)	4.34 (0.85)***	<0.001	4.55 (0.67)	4.11 (1.02)*	0.001	4.05 (0.99)	4.26 (0.95)	
Conflicts openly dealt with	2.61 (1.28)	3.38 (0.95)**	0.001	3.58 (0.98)	3.18 (1.01)*	0.012	3.18 (1.01)	3.31 (1.02)	
MDs and RNs respect opinions	4.26 (0.89)	3.87 (0.86)*	0.010	4.03 (0.86)	3.88 (0.87)		3.44 (0.98)	4.00 (0.82)**	0.001
RNs and NAs respect opinions	3.87 (0.89)	3.77 (0.88)		3.94 (0.76)	3.73 (0.93)		3.21 (0.95)	3.88 (0.85)***	<0.001
Competent coworkers	4.58 (0.62)	4.58 (0.57)		4.51 (0.60)	4.61 (0.57)		4.49 (0.60)	4.60 (0.57)	
Patients' wishes	4.71 (0.53)	4.40 (0.66)**	0.008	4.43 (0.68)	4.44 (0.64)		4.22 (0.73)	4.47 (0.63)*	0.028
Parents' wishes	4.55 (0.57)	4.46 (0.58)		4.53 (0.58)	4.45 (0.57)		4.37 (0.54)	4.49 (0.58)	
Manager helps coworkers	2.39 (1.48)	3.93 (1.02)***	<0.001	4.04 (0.99)	3.65 (1.24)*	0.029	3.66 (1.15)	3.77 (1.20)	
Openness asking questions, learning	4.52 (0.77)	4.48 (0.71)		4.49 (0.71)	4.48 (0.72)		4.21 (0.80)	4.53 (0.69)**	0.008
Practice care as it should be	4.37 (0.81)	4.14 (0.76)		4.14 (0.78)	4.17 (0.77)		3.90 (0.84)	4.21 (0.75)*	0.017
MDs ask RNs	3.70 (0.88)	3.32 (1.04)		3.35 (1.05)	3.37 (1.03)		3.02 (1.13)	3.42 (1.00)*	0.020
RNs ask NAs	3.64 (0.83)	3.66 (0.88)		3.79 (081)	3.61 (0.89)		3.03 (0.93)	3.77 (0.82)***	<0.001
Ethical issues identified	4.13 (0.76)	3.87 (0.90)		3.89 (0.87)	3.90 (0.90)		3.22 (1.04)	4.01 (0.81)***	<0.001
Dealing ethical issues	3.61 (1.20)	3.69 (1.00)		3.57 (0.97)	3.72 (1.04)		2.95 (1.09)	3.81 (0.96)***	<0.001

Abbreviations: MD, medical doctor; NA, nursing assistant; POC, paediatric oncology centre; RN, registered nurse; SD, standard deviation. Differences tested by the Mann‐Whitney U test.

* < 0.05.

** < 0.01.

*** < 0.001.

Another centre (B) scored significantly higher on the three items regarding the immediate manager and the item about conflicts being dealt with openly. In addition to these four items, this centre (B) had significantly higher scores on one item and no significantly lower values (Table 4).

A third centre (C) had significantly lower scores than the other POCs on 10 items, including items concerning the relationship between HCP (n = 5), team interactions (n = 1), patient wishes (n = 1), the possibility of practicing care as they think it should be practiced (n = 1), and identifying and dealing with ethical issues (n = 2). This centre did not have significantly higher values on any of the items (Table 4) and had a significantly (*P* ≤ 0.001) lower total score (mean 3.61) than the other centres.

A significant positive correlation between the perception of the possibility of practicing ethically good care and the per person mean values the rest of the items was found in all centres (A: *r* = 0.55, *P* = 0.002; B: *r* = 0.51, *P* = 0.005; C: *r* = 0.38, *P* = 0.016; D: *r* = 0.47, *P* < 0.001; E: *r* = 0.36, *P* = 0.009; F: *r* = 0.54, *P* < 0.001).

## 4 DISCUSSION

The key results of this study of HCP's perceptions of the ethical climate in Swedish paediatric oncology were as follows: (a) MDs reported that they asked RNs for their opinions on treatment whilst RNs did not have this perception to the same extent; (b) MDs experienced the ethical climate as being more positive than RNs and NAs; (c) NAs with less than 5 years of experience reported, to a larger extent than those with more experience, that hospital guidelines helped them; (d) at the POC with the significantly lowest values concerning immediate manager, no significant correlation with the other items was found; and (e) at the POC with the poorest ethical climate, HCP also reported the lowest possibility of practicing ethically good care. These results will be discussed below.

In this study the item on MDs asking RNs for their opinions regarding treatment was one of the items that had the lowest overall value. This item had been removed from the HECS‐S but was reintroduced in the Swedish HECS‐S.^16^ One reason for removing this item could be that MDs actually do not ask RNs about treatment issues but rather about nursing issues. However, in this study, MDs scored significantly higher than RNs on this item. Furthermore, this was the only item in which a gender difference could be identified as male MDs perceived that MDs ask RNs to a greater extent than female MDs. Thus, MDs, particularly male MDs, had the perception that they asked RNs for their opinions on treatment whilst RNs did not have this perception to the same extent. This view of the RNs is congruent with the results of a qualitative study in which conflicting perspectives emerged as an ethical concern, and nurses felt that they could not influence medical decisions.^2^

MDs stand out in this study as the group with higher scores compared with the other two groups as they scored significantly higher values than RNs and/or NAs on 12 out of the 21 items. Significant differences between NAs and RNs were only identified in three items. This can be compared with the results of a previous study conducted in one hospital where MDs and NAs had similar scores, whilst RNs stood out as the group with lower scores than the other two groups.^13^ Similarly, in a study from the United States, RNs experienced a poorer ethical climate than MDs.^12^ This could partly be explained by MDs having procedures for discussing difficult issues with colleagues and the multidisciplinary team and rating this collaboration as being more favourable than RNs.^12^ Moreover, support from colleagues has been identified as a main contributory factor for MDs' resilience.^19^ This could also explain why, in a Swedish paediatric oncology study, MDs had a significantly lower total moral distress score compared with RNs.^20^

The only significant difference identified between years of experience was that NAs with less than 5 years of experience scored higher on the item on “guidelines help me” than NAs with more experience. Initially, the results indicated a difference between years of experience on the items concerning identifying and dealing with ethical issues. However, this difference rather related to the significantly higher scores on these items amongst MDs, of whom almost all had more than 5 years of experience. These high scores could be explained by the MDs being “forced” to deal with ethical issues because studies have shown that they assume a considerable amount of responsibility^3^ and also feel great uncertainty in difficult decisions.^2^

In the present study, one of the centres (A) had significantly *lower* values on the three items regarding the immediate manager, with no significant correlation to the other items. This indicates that a negative perception of the immediate manager does not necessarily mean that the ethical climate is poor. Furthermore, another centre (C) had significantly lower values on 10 items (but not regarding the immediate manager) than the other centres including centre A with low values regarding the immediate manager. This could be seen to contrast with Silvermans’^21^ claim that management support is needed to create an ethical climate. However, items on manager support in the HECS‐S concern the immediate manager being involved and available to discuss difficult patient care situations. The results of the present study could be related to Swedish working culture and the role of the immediate manager, who is usually not involved in direct patient care. Having high values on the other aspects of the ethical climate could still be the result of previous good leadership/management if; for example, they supported routines that enhanced collaboration and dialogue between coworkers. As previously suggested, management that supports ethical behaviour generates positive organisational outcomes.^5^ The centre (C) that had significantly lower scores on 10 items also had the lowest overall score. This could relate to the fact that many items in the HECS‐S concern the relationship between the different professions and team interaction. However, we would argue that the fact that this centre (C) also had the lowest values regarding the possibility of practicing care as it should be practiced could be seen as an indication that the ethical climate at this centre will negatively influence patient care. Also, in a previous study, a positive perception of interprofessional trust was related to the possibility of practicing ethically good care.^13^

Obvious strengths of this study are the nationwide and multi‐professional coverage, as well as the high response rate. The latter limits the risk of nonresponse bias and increases the generalisability, not only to international paediatric oncology but also to other highly specialised paediatric settings.

## 5 CONCLUSIONS

Because paediatric oncology entails difficult ethical issues, an ethical climate is crucial for preventing moral distress and staff turnover, as well as for the quality of patient care. Differences in the perception of the ethical climate have been shown between professional groups as MDs perceive the ethical climate to be more satisfying than the two other health‐care professions (RNs and NAs). Good relationships between the different professions and an open dialogue within the health‐care team is key to achieving an ethical climate. Furthermore, as this research has demonstrated, there are apparent differences between centres, and the perception of the possibility of practicing ethically good care seem to be an indicator of the other aspects of the ethical climate in paediatric oncology. Interestingly, a negative perception of the immediate manager does not necessarily mean that the ethical climate is poor.

### 5.1 Study limitations

A potential limitation of the HECS‐S is that the items concerning the immediate manager do not fully capture this aspects of the ethical climate. It is reasonable to assume that rather than discussing patient‐care issues, the role of the immediate manager is to provide opportunities for HCP to deliberate on ethical issues and collaborate interprofessionally. Furthermore, many statistical tests were performed, increasing the risk of mass significance.

### 5.2 Clinical implications

The understanding of the hospital ethical climate from a multidisciplinary perspective in paediatric cancer care could facilitate the formulation of plans for organisational improvments. The knowledge from this study motivates actions to promote a good ethical climate by supporting interprofessional collaboration and providing ethics support in identifying and dealing with ethical issues, especially for RNs and NAs. Furthermore, it is important to deal with the issues in the interprofessional collaboration due to the descrepancies in perceptions on MDs asking RNs for their opinions on treatment.
